# Unusual cause of gastric outlet obstruction: giant gastric trichobezoar: a case report

**DOI:** 10.1186/1757-1626-1-399

**Published:** 2008-12-16

**Authors:** Ibrahim Yetim, Orhan Veli Ozkan, Ersan Semerci, Recep Abanoz

**Affiliations:** 1 Mustafa Kemal University, Faculty of Medicine, Department of General Surgery, Antakya/Hatay, Turkey; 2 Bafra State Hospital, Bafra/Samsun, Turkey

## Abstract

**Background:**

Trichobezoars are caused by hair ingestion. The usual presentation of a trichobezoar is with early satiety and malnutrition. Obstructive symptoms and manifestations of gastric outlet obstruction may occur. The diagnosis may be suspected in young females with malnutrition, who have a history of trichophagia.

**Case presentation:**

We report a case of 12-year-old female admitted to the emergency room for abdominal pain. On physical examination, she was cachectic and an epigastric mass was palpated. An exploratory laparotomy was conducted. A giant trichobezoar was palpated in the stomach and was removed through an anterior gastrostomy.

**Conclusion:**

There were no complications postoperatively and the patient was referred to a psychiatrist.

## Background

Bezoars are incompletely digested food or fibrous materials that may cause intestinal obstruction over the time. Common clinical symptoms include abdominal pain, nausea, vomiting, weight loss [1]. Trichobezoars are seen in case of large amount of hair ingestion. Trichobezoars are more common in paediatric age than adults with up to 90% occurring in girls [2]. In this report, we describe a 12-year-old girl with obstructing trichobezoar of stomach and duodenum.

## Case report

**Figure 1:**
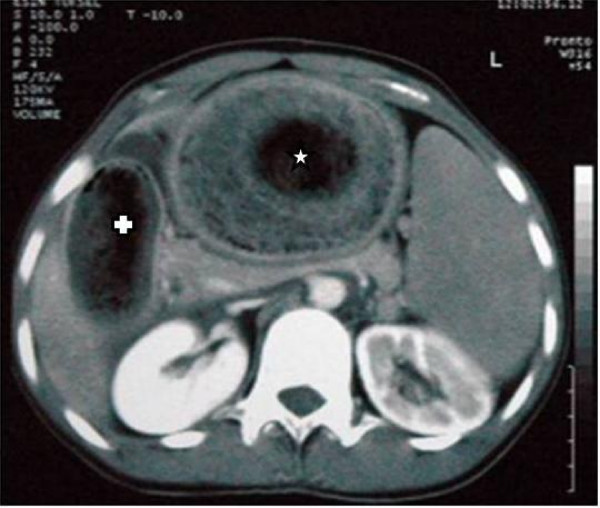
**Computed tomogram of the Abdomen**. Trichobezoar seen on Stomach (☆) and duodenum (✞)

**Figure 2:**
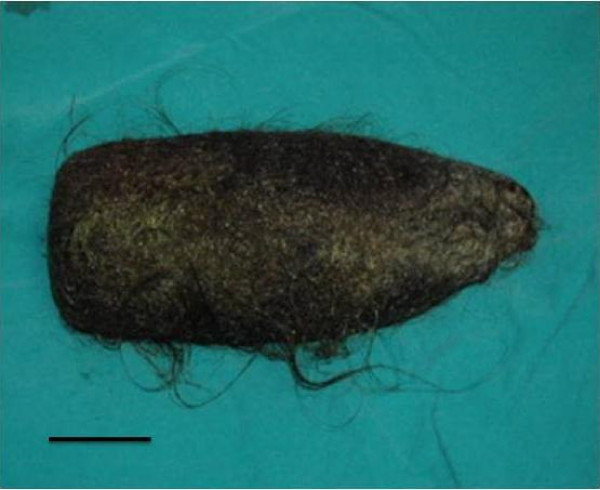
**Trichobezoar extracted from stomach with an approximate length 22 cm (Black bar is shown approximately 5 cm length)**.

A 12 year old girl was admitted to our emergency department with 5 days history of abdominal pain and non-bilious vomiting. On physical examination, patient was cachectic and an epigastric mass was palpated. Biochemical results were normal except mild anaemia. X-ray depicted that stomach was dilated with no air-fluid level. Ultrasonography (USG) revealed hyperechoic mass on the epigastric region. On the computed tomography (CT) appearance, there was a heterogenous lesion from stomach to horizontal duodenal part that causes distation with circumcised air trapping (Figure 1). Patient had history of trichophagia for a long time. Patient underwent laparotomy. Upper midline incision was performed. A giant trichobezoar was removed through an anterior gastrostomy. This mass extended to proximal part of duodenum. The mass weighed 125 g and measured 8.5 × 22 cm (figure 2). There was no complication postoperatively. Patient was discharged a week later.

## Discussion

The term bezoar was derived from the Persian word 'panzeh' meaning 'antidote' [3]. In ancient times, bezoars were considered as a protection against poisoning. They are divided into three categories according to components; phytobezoars (plant material), trichobezoars (hair) and lactobezoars (milk) [4].  Phytobezoars are generally found in patients with history of gastric surgery. Lactobezoars are exclusively found in infants. Prematurity and concentrated formulas are leading causes of lactobezoars. Trichobezoars are caused by ingestion of high amount of hair over many years. They are formed typically in stomach and they may enlarge leading to gastric outlet obstruction [5,6] The cause of hair ingestion may be associated with mental retardation, pica or trichotillomania which is a behavioural disturbance characterized by the compulsive urge to pull one's hair and eat it. Up to 90% of all trichobezoars occur in girls younger than 20 years old [2]. Males are rarely affected. Trichobezoar have a special type called 'Rapunzel Syndrome'. In that syndrome gastric trichobezoars could have a long tail that can extent to ileoceal valve [7,8].

Common presenting symptoms are abdominal pain, nausea, vomiting, weight loss, malnutrition, hematemesis, diarrhoea or constipation. On physical examination epigastric mass may be palpated. Alopecia may also be present due to trichotillomania.

USG and CT imaging features are helpful in diagnosis. On the USG, bright echogenic band and shadow over the left upper quadrant may exist. CT demonstrates heterogenous, mottled intraluminal mass with low attenuation and air trapping [9].

Phytobezoars may be digested enzimatically but trichobezoars are generally resistant to that treatment. Small gastric bezoars can be removed endoscopically. Large gastric trichobezoars can be removed either endoscopically or by laparotomy. In our case, a giant bezoar had caused duodenal obstruction. Small intestinal obstruction is rare but small intestine examination is important for ensuring about any other mass remnant.

Since recurrences could occur, the patient must be directed to psychiatric follow-up after the operation.

## Consent

Written informed consent was obtained from the patient for publication of this case report and accompanying images. A copy of the written consent is available for review by the Editor- in – Chief of this Journal.

## Competing interests

The authors declare that they have no competing interests.

## Authors' contributions

IY and RA analysed and interpreted the patient data. OVO and ES performed the literature review, and was a major contributor in writing the manuscript. OVO and IY performed the final editing of the manuscript. All authors read and approved the final manuscript.
